# Isolation, In Vitro Antioxidant Capacity, Hypoglycemic Activity and Immunoactivity Evaluation of Polysaccharides from *Coriandrum sativum* L.

**DOI:** 10.3390/antiox14020149

**Published:** 2025-01-27

**Authors:** Weiwei Jin, Huan Zhou, Haijun Zhao, Yue Pei, Fengxian Su, Yan Li, Tao Luo

**Affiliations:** 1 Institute of Food Science, Wenzhou Academy of Agricultural Science, Wenzhou 325006, China; wwjin2003@163.com (W.J.); zhouhuan@wzvcst.edu.cn (H.Z.); 18293997473@163.com (H.Z.); 17793671534@163.com (Y.P.); sufengxian@wzvcst.edu.cn (F.S.); liyan@wzvcst.edu.cn (Y.L.); 2 Southern Zhejiang Key Laboratory of Crop Breeding, Wenzhou 325006, China; 3 College of Life Science and Engineering, Lanzhou University of Technology, Lanzhou 730050, China; 4 College of Horticulture, South China Agricultural University, Guangzhou 510642, China

**Keywords:** coriander polysaccharides, antioxidant activity, hypoglycemic activity, immunoregulation, anti-tumor activity

## Abstract

Coriander (*Coriandrum sativum*) is a classical medicinal and edible herb as well as a spice, but the physicochemical and biological properties of its polysaccharides have not been fully studied. In this study, the polysaccharides were extracted using an ultrasonic-assisted method and purified from fresh coriander, and then the coriander polysaccharide (CSP) fraction was separated using an agarose gel Q-Sepharose Fast Flow column. The total sugar content, protein content and monosaccharides composition of CSPs were determined using a phenol–sulfuric acid method, Coomassie Brilliant Blue method and HPLC. The structural characterization was detected using ultraviolet spectrophotometry and FT-IR spectroscopy. DPPH and ABTS free radicals were used to explore their antioxidant activities, while the inhibitory abilities of α-amylase and α-glucosidase were used to evaluate their hypoglycemic activity. After that, the immunomodulatory and antitumor activities were investigated using macrophage RAW264.7 and HepG2 cells as the targets. The results showed that the total sugar and protein contents of CSPs were 66.90 ± 1.44% and 1.06 ± 0.32%, respectively. CSPs were mainly composed of fucose, rhamnose, arabinose, galactose, glucose, galacturonic acid and glucuronic acid, with a molar ratio of 1.13:15.11:9.60:25.98:1.55:44.33:2.29, and may be an acidic heteropolysaccharide containing pyran rings, α- and β-glycosidic bonds and glucuronic acid. Results from in vitro experiments of biological activities showed that the IC_50_ of CSPs for scavenging DPPH and ABTS free radicals were 0.759 mg/mL and 1.758 mg/mL, respectively; the IC_50_ values for inhibiting the activities of α-amylase and α-glucosidase were 0.634 mg/mL and 2.178 mg/mL, respectively; the CSPs with a concentration of 25~200 μg/mL showed no obvious toxicity to macrophage RAW264.7, and when treated with 100 μg/mL of CSPs, the relative cell phagocytosis capacity and secreted nitric oxide amount of RAW264.7 were 153.75 ± 12.01% and 133.56 ± 5.37%, respectively; CSPs showed a concentration-dependent ability to inhibit the growth of HepG2 cells within the test concentration of 0.25–2.0 mg/mL. Summarizing the results, due to their excellent antioxidant, immunomodulatory and anti-tumor activities, the coriander acid polysaccharides were expected to show good potential in comprehensive development of food and medicine.

## 1. Introduction

Coriander (*Coriandrum sativum* L.), also named cilantro or Chinese parsley, an annual or biennial herb that belongs to the Umbelliferae/Apiaceae family, was originated from the Mediterranean coast of Europe and introduced into China in the first century BC [1,2]. Because of its particular volatile profile and potential nutritional value, coriander has been one of the most commonly used culinary ingredients around the world [3,4]. In addition, coriander has a long history of being used as a medicine and food homologous plant across regions. The whole plant of coriander has been used as a traditional medicinal herb to treat measles (without adequate eruption) and colds (without sweat) in China [2]. Moreover, coriander can strengthen memory and sleep comfort [5] and has anti-aging [6] and anti-diabetes [7,8] effects, as well as inhibiting both the degranulation of RBL-2H3 cells [9] and formation of polycyclic aromatic hydrocarbons [10]. The active ingredients of coriander are complex, mainly including essential oil [6,11,12,13,14,15], flavonoids [16], polyphenols [8,17], monoterpenes [18], polysaccharides [19,20] and other active chemicals. It is clear that most research focuses on the biological activity of essential oil. However, research on other active ingredients in coriander, such as polysaccharides, is still insufficient.

Polysaccharides represent one of the most important members of the biopolymer family, with low toxicity and good biocompatibility, which are connected by glycosidic bonds to multiple monosaccharides or monosaccharide derivatives [21]. They are widely distributed in various organisms including plants, animals and microbes [22]. Among them, plants are an important source of natural polysaccharides. Many studies have shown that plant polysaccharides have certain antioxidant activities and can remove free radicals such as hydroxyl radicals and superoxide anion radicals [23,24]. 1,1-diphenyl-picrylhydrazyl (DPPH) and 2,2′-azino-bis (3-ethylbenzothiazoline-6-sulfonic acid) (ABTS) methods are frequently used in measuring the antioxidant activities of polysaccharides. Plant polysaccharides also showed certain immune activities, by activating macrophage immune responses, including increasing the production of reactive oxygen species and enhancing the secretion of cytokines and chemokines [25]. However, the bioactivities of plant polysaccharides were affected by extraction and purification methods, because they may change the molecular and composition of monosaccharides [26].

The significant biological activity of polysaccharides derived from various Chinese herbal medicines has attracted more and more attention in recent decades. Among them, polysaccharides from medicinal and edible homologous plants have been widely confirmed to have a broad spectrum of antioxidant and medicinal biological effects [26,27,28,29]. Polysaccharides from *Polygonatum kingianum* could promote macrophage secretion of nitric oxide (NO) and cytokines (TNF-α, IL-6) by activating the NF-κB signaling pathway, demonstrating immunomodulatory activity [27]. HepG2 cells treated with dandelion leaf polysaccharides displayed clear apoptotic morphology, including cell volume decreases and cytoskeleton breakdown, indicating the potential of the polysaccharide as an anticancer agent [28]. Coriander is also a classical medicinal and edible herb. Polysaccharides extracted from coriander seeds were composed of arabinose, rhamnose, xylose, mannose, fructose, galactose and glucose. They could prevent cadmium-induced oxidative damage and have a protective effect on cadmium-induced liver cell toxicity, by significantly reducing the production of MDA and lowering the levels of CAT and SOD enzymes [19]. However, the polysaccharides in reference [19] were a lack of further purification, such as elution with water or NaCl solution on a gel column. The polysaccharides from fresh coriander were composed of arabinose, rhamnose, galactose, glucose and galacturonic acid [20], which differed from the polysaccharides in coriander seeds [19]. The sugar residues of polysaccharides from fresh coriander were α-L-Araf-(1→, →6)-β-D-Galp-(1→, →4)-α-GalpA-(1→ and →2, 4)-α-Rhap-(1→) [20], but there was no report about sugar residues in reference [19]. The polysaccharides from fresh coriander could suppress H22 tumor growth by inducing apoptosis and S-phase arrest, which was similar to the mechanism of dandelion leaf polysaccharides in HepG2 cells [20,28]. However, to our knowledge, only these two papers have reported on the polysaccharides in coriander. More information about the polysaccharides from coriander needs to be explored.

In this study, the antioxidant, hypoglycemic, immunomodulatory and antitumor biological activities of polysaccharides isolated from coriander were systematically investigated. The results are expected to provide theoretical basis for the comprehensive development of coriander in the fields of medicine and consumption.

## 2. Materials and Methods

### 2.1. Chemicals

Sulfuric acid, trichloromethane and 3,5-Dinitrosalicylic acid (DNS) (analytical pure grade, Zhejiang Zhongxing Chemical Reagent Co., Ltd., Lanxi, China); ethanol (analytical pure grade, Anhui Ante Food Co., Ltd., Suzhou, China); anhydrous glucose (analytical pure grade, Xilong Scientific Co., Ltd., Shantou, China); sodium chloride, n-butyl alcohol, sodium dihydrogen phosphate and disodium hydrogen phosphate (analytical pure grade, Sinopharm Chemical Reagent Co., Ltd., Shanghai, China); L-Ascorbic acid (Vc), 4-Nitrophenyl-β-D- glucopyranoside (PNPG, Hefei Qiansheng Biotechnology Co., Ltd., Hefei, China); DPPH (>97.0%(HPLC), Tokyo Chemical Industry (TCI) Co., Ltd., Shanghai, China); ABTS (≥98.0%, Cool Chemistry (Coolbjc) Co., Ltd., Hefei, China); acarbose, α-amylase, α-glucosidase (Shanghai Lanji Technology Development Co., Ltd., Shanghai, China); RAW264.7 (CL-0190) and HepG2 (CL-0103) (Wuhan Pricella Biotechnology Co., Ltd., Wuhan, China); Neutral Red Staining Solution Kit and Lipopolysaccharides (LPSs, Beyotime Biotechnology Co., Ltd., Shanghai, China); CCK8 (APExBIO Technology Co., Ltd., Shanghai, China); Griess Reagent (Beijing Solarbio Science & Technology Co., Ltd, Beijing, China); potassium bromide (KBr, spectroscopically pure grade, Sigma-Aldrich Co., Ltd., Shanghai, China); trifluoroacetic acid (TFA), sodium hydroxide (NaOH), sodium acetate (NaAc, chromatographic grade, Shanghai Anpel Experimental Technology Co., Ltd., Shanghai, China); Q Sepharose Fast Flow (Cytiva Co., Ltd., Shanghai, China).

### 2.2. Extraction and Purification of CSPs from Coriander

The extraction and purification of polysaccharides were performed according to [30], with some modification. Fresh coriander was purchased from Yonghui supermarket on June 2022 (Wenzhou, Zhejiang, 27.91″ N, 120.71″ E) and was dried at 50 °C to a constant weight, ground into powder and placed in a 2 L beaker. The powder was mixed with ten times the amount of anhydrous ethanol, and the mixture was refluxed at 70 °C for 3 h until it was almost colorless to remove small molecular substances such as pigments and fats. It was then filtered, and the filter residue was collected and air-dried for later use.

The dried powder was weighed and mixed with distilled water in a ratio of 1:30. The water solution was subjected to ultrasonication (power, 240 W) at 60 °C twice (30 min each time) to extract polysaccharides. The extracted solutions were combined and condensed with evaporation. The concentrated material was mixed with 4 times the amount of anhydrous ethanol, placed overnight at 4 °C and centrifuged, and the sediment was then collected. The sediment was redissolved and purified by removing protein and color. The Sevag method (Sevag solution composed of n-butanol and chloroform (1:4, *v*/*v*)) was used to remove protein, and a D354FD macroporous resin column (Ningbo Zhengguang Co., Ltd., Ningbo, China) was used for decolorization. Then, polysaccharides were dialyzed in 7 kDa Mw cut-off dialysis bags (Hunan Yibo Biological Co., Ltd., Changsha, China) under running tap water for 48 h, followed by the second dialysis in distilled water for 24 h. After that, crude coriander polysaccharides were obtained through vacuum concentration and freeze-drying.

A measure of 0.120 g of the crude coriander polysaccharides was weighted and dissolved in 40 mL distilled water, followed by centrifugation at 5581 g/min for 10 min. The supernatant was collected and filtered through a 0.22 μm aqueous microporous membrane. Using a Q Sepharose^TM^ Fast Flow column (2.5 × 60 cm), gradient elution was performed sequentially with NaCl solution ranging from 0 to 1.0 mol/L. The flow rate was controlled at 1.0 mL/min using a DHL-2 computer constant flow pump (Shanghai Jingke Co., Ltd., Shanghai, China), with 10 mL collected in each tube and 50 tubes collected in each gradient. The phenol sulfuric acid method was used for tracking and detection, and the elution curve was plotted [31,32]. The main components were collected for dialysis, concentration and freeze-drying, and the coriander polysaccharides were obtained (abbreviated as CSPs).

### 2.3. Total Sugar Content and Protein Content Analysis in the CSPs

The phenol sulfuric acid method was used for determination of the total sugar content [31,32]. Glucose solutions with concentrations of 0.01, 0.03, 0.05, 0.07 and 0.09 mg/mL were prepared, and 2 mL of each solution was taken into a test tube. A measure of 1 mL of 5% phenol solution was added, and after mixing evenly with a KF-NP-28S vortex analyzer (Hangzhou Yanhe Equipment Co., Ltd., Hangzhou, China), 5 mL of sulfuric acid was immediately added. The mixture was naturally cooled to room temperature for detection, and the distilled water was used as a blank. Three parallel replicates were used for each measurement. The results were plotted with the glucose concentration as the horizontal axis and OD_490_ as the vertical axis to form a standard curve. The absorbance value of 0.05 mg/mL of CSPs was measured, and the total sugar content in CSP was calculated based on the regression equation of the glucose standard curve.

The protein content in the CSPs was determined using the Coomassie Brilliant Blue method, with bovine serum albumin (BSA) standard solution as the control [31]. The standard BSA solutions with concentrations of 0.02, 0.04, 0.06, 0.08 and 0.1 mg/mL were prepared. Six groups of test tubes were prepared, with three repeats in each group. Measures of 1 mL of BSA solution and 5 mL of Coomassie Brilliant Blue G-250 solution were added to each tube, and the tubes were incubated at 30 °C for 30 min. After sufficient cooling, the OD_595 nm_ of each tube was measured. Three parallel replicates were used for each measurement. The BSA standard curve was plotted based on the concentration of BSA and OD_595 nm_. The absorbance value of 0.05 mg/mL of CSPs was measured, and the protein content in CSPs was calculated based on the BSA standard curve regression equation.

### 2.4. UV–Vis Spectrometry Analysis of CSP

The UV/visible spectrophotometer 1800-DS2 (MAPADA, Shanghai, China) was used to record the absorbance of CSP solution (0.1 mg/mL) with the range of 200 to 800 nm [27].

### 2.5. FT-IR Spectroscopy Analysis of CSP

The dried CSP powder was mixed with KBr powder with a ratio of 1:100 and then pressed into a tablet for FI-TR assay on a Nicolet iS-10 FT-IR spectrometer in the range of 400 to 4000 cm^−1^ (Thermo Fisher Scientific Co., Ltd., Shanghai, China) [27].

### 2.6. Monosaccharide Composition Analysis of CSP

The monosaccharide composition was determined according the method from reference [27], with slight modifications. An appropriate amount of CSPs was dissolved with 1 mL of 2 M TFA and reacted at 120 °C for 2 h, which was then blow dried with nitrogen gas. Methanol was added three times to remove excess TFA, and nitrogen gas was used for drying each time. Then, samples were dissolved in sterile water, filtered through a 0.22 μm microporous membrane and transferred to a chromatography bottle for testing.

The samples were analyzed on an ICS 5000+ ion chromatography system (Thermo Fisher Scientific Co., Ltd., Shanghai, China) with an electrochemical detector and a Dionex^TM^ CarboPac^TM^ PA20 column (150 mm × 3.0 mm, 10 μm) (Thermo Fisher Scientific Co., Ltd., Shanghai, China). The mobile phase was a mixture of A (pure water), B (0.1 M NaOH) and C (0.2 M NaAc). The elution gradient was set as follows: 0 min A/B/C (95:5:0, *v*/*v*), 26 min A/B/C (85:5:10, *v*/*v*), 42 min A/B/C (85:5:10, *v*/*v*), 42.1 min A/B/C (60:0:40, *v*/*v*), 52 min A/B/C (60:40:0, *v*/*v*), 52.1 min A/B/C (95:5:0, *v*/*v*), and 60 min A/B/C (95:5:0, *v*/*v*). The flow rate was 0.5 mL/min, and the column temperature was maintained at 30 °C.

### 2.7. In Vitro Antioxidant Capacity Analysis of CSP

The DPPH radical scavenging rate was determined according to the method from reference [33], with slight modifications. A measure of 0.1 mmol/L DPPH solution with anhydrous ethanol, the CSP and Vc (set as positive control) solutions of different concentrations were prepared. The reagents were added into a test tube according to Table 1, then mixed well with a vortex analyzer, and the reaction in each tube was performed in the dark for 30 min. Three parallel replicates were used for each group. The absorbance value of the mixed solution at 517 nm was detected, and the DPPH radical scavenging rate was calculated using Equation (1). The result was expressed as a percentage (%).

(1) $$DPPH\;radical\;scavenging\;rate\left(\%\right)=\frac{A3-(A1-A2)}{A3}\times 100$$

where A1 is the absorbance value of the extracted sample reacted with DPPH, A2 is the background absorbance value of the extracted sample (without DPPH), and A3 is the absorbance value of the blank sample (only DPPH without sample).

The ABTS free radical scavenging rate was determined according to the method reported in reference [33] with slight modifications. The ABTS solution with phosphate-buffered saline (PBS), the CSP and Vc (set as positive control) solutions of different concentrations were prepared. The reagents were added into a test tube according to Table 2 and mixed well with a vortex analyzer, and the reaction in each tube was performed for 20 min with the absence of light. Three parallel replicates were performed for each group. The absorbance value of the mixed solution at 734 nm was detected, and the ABTS free radical scavenging rate was calculated using Equation (2). The result was expressed as a percentage (%).

(2) $$ABTS\;free\;radical\;scavenging\;rate\left(\%\right)=\frac{A3-(A1-A2)}{A3}\times 100$$

where A1 is the absorbance value of the extracted sample reacted with ABTS, A2 is the background absorbance values of the extracted sample (without ABTS) and A3 is the absorbance values of the blank sample (only ABTS without sample).

### 2.8. In Vitro Hypoglycemic Activity Analysis of CSP

#### 2.8.1. Effects of CSPs on α-Amylase Inhibitory Activity

The α-amylase inhibitory activity was determined according to the method in reference [34], with slight modifications. The α-amylase solution (25 U/mL) and starch solution (1%) were prepared, as were different concentrations of acarbose (the positive control) and CSP solutions (using 0.1 mol/L PBS to dissolve). The reagents were added to a test tube for reaction according to Table 3. Three parallel replicates were used for each group. The OD_540nm_ of each group was measured, and the α-amylase inhibition rate was calculated using Equation (3). The result was expressed as a percentage (%).

(3) $$Inhibition\;rate\;of\;\alpha \text{-}amylase\left(\%\right)=\frac{A3-(A1-A2)}{A3}\times 100$$

where A1 is the absorbance value of DNS reacted with reducing sugar released by α-amylase inhibited by CSPs or acarbose, A2 is the background absorbance values of the extracted sample (without α-amylase) and A3 is the absorbance values of the blank sample (only α-amylase without CSPs or acarbose).

#### 2.8.2. Effects of CSPs on α-Glucosidase Inhibitory Activity

The α-glucosidase inhibitory activity was determined according to the method in reference [34], with slight modifications. Measures of 1 U/mL of α-glucosidase solution, 5 mmol/L of PNPG and 0.2 mol/L of sodium carbonate solution using 0.1 mol/L of PBS as a solvent were prepared, as were different concentrations of acarbose (used as positive control) and CSP solutions. The reagents were added into a test tube for reaction according to Table 4. Three parallel replicates were performed for each group. The OD_405 nm_ of each tube was measured, and the inhibition rate of α-glucosidase was calculated using Equation (4). The result was expressed as a percentage (%).

(4) $$Inhibition\;rate\;of\;\alpha \text{-}glucosidase\left(\%\right)=\frac{A3-(A1-A2)}{A3}\times 100$$

where A1 is the absorbance value of DNS reacted with reducing sugar released by α-glucosidase inhibited by CSPs or acarbose, A2 is the background absorbance value of the extracted sample (without α-amylase), and A3 is the absorbance value of the blank sample (only α-glucosidase without CSPs or acarbose).

### 2.9. Immunoactivity Evaluation of CSP

#### 2.9.1. Effects of CSPs on RAW264.7 Cell Viability

The CCK8 method was used to analyze the effects of polysaccharides on RAW264.7 cell viability [27,35]. A measure of 100 μL of the RAW264.7 cell suspension in logarithmic growth phase was added to a 96-well culture plate and cultured for 24 h with the conditions of 37 °C and 5% CO_2_. A measure of 100 μL of CSPs with different concentrations (final concentrations of 0.25, 0.5, 1.0, and 2.0 mg/mL) was added to the wells. A measure of 100 μL of DMEM culture medium was used as a control group, and 3 parallel tests were set in each group. After culture, 10 μL of CCK8 was added to each well. After further incubation for 1 h, the OD_450nm_ of each group was measured using a Multiskan FC microplate reader (Thermo Fisher Scientific Co., Ltd., Shanghai, China). The results (cell viability) were expressed as a percentage of OD_450nm_ of each group compared with that of the corresponding control.

#### 2.9.2. Effects of CSPs on Phagocytosis Activity of RAW264.7 Cells

The neutral red method was used to detect the effects of CSPs on the phagocytic activity of RAW264.7 cells [27,35]. DMEM medium and LPSs (1 μg/mL) were used as blank and positive controls, respectively. The experimental group was set with low (25 μg/mL), medium (50 μg/mL) and high (100 μg/mL) final concentrations of CSPs, with three parallel repeats at each concentration. The 96-well plate was cultured under the conditions of 37 °C and 5% CO_2_ for 24 h, and the culture medium was then discarded. A measure of 20 μL of neutral red staining solution was added to each well, followed by incubation for 2 h. After washing with PBS three times, 200 μL of neutral red detection lysate was added to each well. The lysate was incubated on a shaker at room temperature for 10 min, and the OD_540nm_ of each group was measured using a Multiskan FC microplate reader. The results (phagocytosis rate) were expressed as a percentage of the OD_540nm_ of each group compared to that of the corresponding control.

#### 2.9.3. Effects of CSPs on NO Secretion in RAW264.7 Cells

The Griess assay kit was used to detect the effects of CSPs on NO secretion in RAW264.7 cells [27]. The supernatant of each group of cell cultures in Section 2.8.1 was collected, with 3 replicates in each group. A measure of 100 μL of the supernatant of each group was added into the 96-well culture plate, and the reagents were added following the instruction of the kit. All of the solutions were mixed well and reacted at room temperature for 10 min. The OD_550nm_ of each group was detected and compared with that of the control group to analyze the NO secretion of RAW264.7 cells in each group.

#### 2.9.4. Effects of CSPs on Proliferation of HepG2 Cells

The CCK8 method was used to analyze the effects of polysaccharides on HepG2 cell proliferation [28]. A measure of 100 μL of the HepG2 cell suspension in logarithmic growth phase was added to a 96-well culture plate and cultured for 24 h with the conditions of 37 °C and 5% CO_2_. A measure of 100 μL of CSPs with different concentrations (final concentrations of 0.25, 0.5, 1.0 and 2.0 mg/mL) was added to the wells. A measure of 100 μL of DMEM culture medium was used as a control group, and 3 parallel tests were set in each group. After culturing, 10 μL of CCK8 was added to each well. After further incubation for 1 h, the OD_450nm_ nm of each group was measured and compared with that of the control group to analyze the cell viability of HepG2 cells in each group.

### 2.10. Statistics

All trials were conducted in three replicates. SPSS 19.0 (IBM, Armonk, NY, USA) was used for data analysis, and the significance of difference between groups was evaluated using one-way analysis of variance (ANOVA) and the least significant difference (LSD) method. The data were plotted using Origin 2021 (OriginLab Corporation, Northampton, MA, USA).

## 3. Results and Discussion

### 3.1. Isolation and Purification of Polysaccharides from Coriander

Traditional water extraction is a common method for extracting polysaccharides from plants for its easy operation and safety [36]. The ultrasound technique could help to disrupt the cell wall and cell membrane of plants and extract more polysaccharides [30,36]. The effectiveness of extraction depends on various factors, such as the liquid–solid ratio, ultrasound powder, and length and temperature of extraction. In our research, crude polysaccharides were extracted from the whole herb of fresh coriander using ultrasonic-assisted hot water extraction.

**Figure 1:**
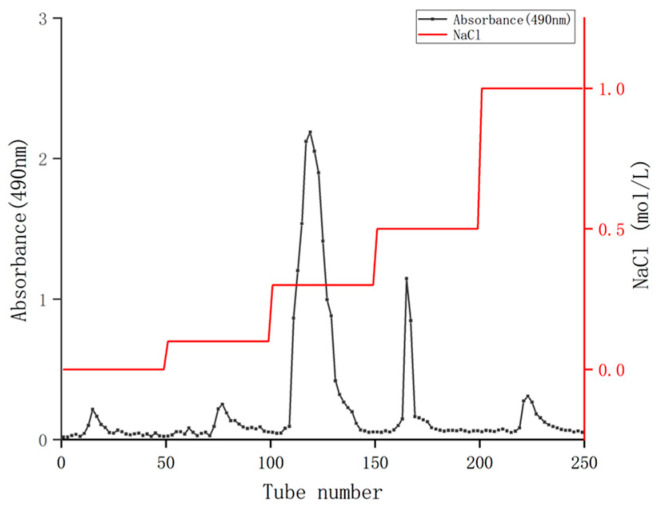
Q Sepharose ^TM^ Fast Flow elution curve of coriander polysaccharides.

The crude polysaccharides of coriander were isolated and purified using a Q Sepharose^TM^ fast low column to remove foreign substances such as proteins (Figure 1). After washing with different concentrations of NaCl solution ranging from 0 to 1.0 mol/mL, five elution peaks were obtained. Among them, the elution area of 0.3 mol/mL NaCl was the largest, indicating that the polysaccharide content was the highest. Therefore, the 0.3 mol/mL NaCl eluent was combined, concentrated, dialyzed, and freeze-dried, and the polysaccharides of coriander (CSPs) was obtained for subsequent activity experiments. The final yield of CSPs obtained from fresh coriander herb was 1.87%, which was lower than that of the whole herb of coriander originated from Jiangsu Province of China (4.56%) reported in the reference [20]. It should be mentioned that the 4.56% polysaccharide yield in [20] was purified with ultra-pure water eluent, while in the current study, CSPs were purified with NaCl eluent (with high ionic strength), which may further promote the purification of polysaccharides by affecting their solubility or charge state. Therefore, washing with NaCl solutions might be one reason for this low yield. The yield of CSPs was also lower than that of coriander seeds (9.10%), which might be because that coriander seed polysaccharides had not been further purified, that is, eluted with water or NaCl solution on a gel column [19]. Another possible reason was that the extraction rate of polysaccharides varies due to different organs. Plant seeds usually have richer nutrient contents.

### 3.2. Physicochemical Properties of CSP

The total sugar and protein contents of CSPs were detected. The glucose standard curve fitting equation for sugar detection was Y = 14.25X + 0.0553 (R^2^ = 0.9997), while the BSA standard curve regression equation for protein detection was Y = 6.2405X − 0.0013 (R^2^ = 0.999). The total sugar and protein content in CSPs was 66.90% ± 1.44 and 1.06% ± 0.32, respectively. The total sugar content was similar to that of the polysaccharide content from coriander seeds (62.2%) [19] and was much lower than that of polysaccharides from fresh coriander (90.16%) [20]. The difference might be due to the different extraction method. The material was defatted with petroleum ether that had better permeability and solubility than ethanol and was extracted at a higher temperature (81 °C) in reference [20]. The protein content was a little higher than that of coriander polysaccharides (0%, 0.62%) in reference [19,20], respectively, but lower than that of polysaccharides from garlic bolt (2.41%) [37]. According to the report, the protein removal rate of the Sevag method was high, but the loss of polysaccharides was severe [31], which also might be one of the reasons for the low CSP yield (1.87%).

### 3.3. UV–Vis Spectrometry Analysis of CSP

**Figure 2:**
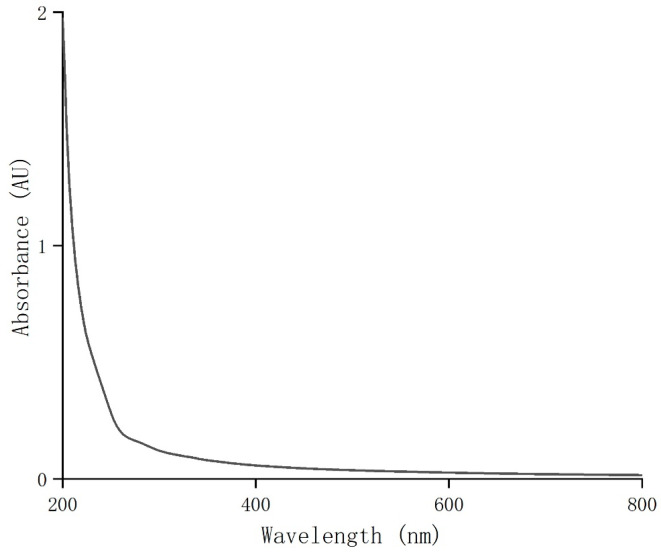
UV–visible spectrum of CSPs.

UV full-wavelength scanning was performed on the purified coriander polysaccharides (CSPs) at 200–800 nm. The result showed that CSPs had weak UV absorption around 260 nm and 280 nm (Figure 2). Combined with the results of the total sugar and protein contents, this indicated that the purified coriander polysaccharides did not contain or contained very few impurities, proteins or peptides, indicating a high purity of CSPs in this work.

### 3.4. FT-IR Spectroscopy Analysis of CSP

**Figure 3:**
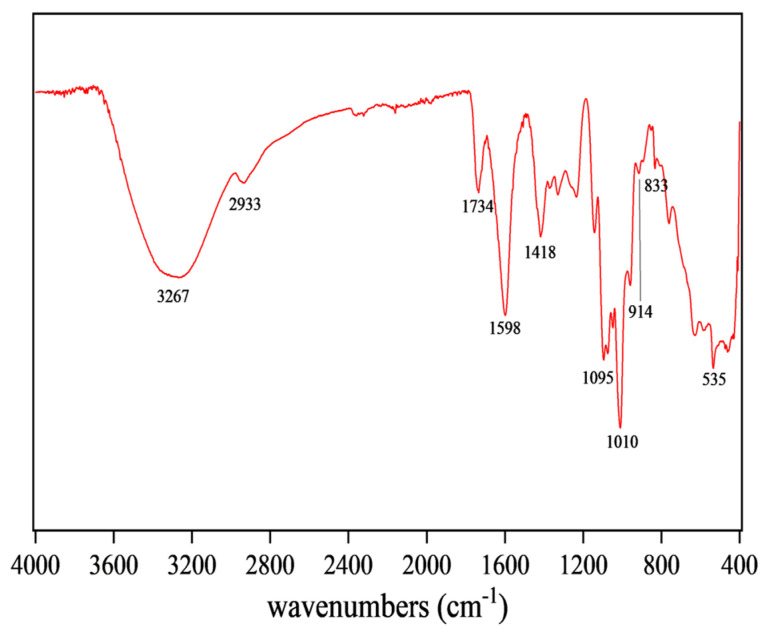
FT-IR spectroscopy of CSPs.

FT-IR can reflect the type of functional groups in compounds, so the structure of CSPs could be inferred from Figure 3. The broad and intense peak at 3267 cm^−1^ was attributed to O-H stretching vibration, indicating that the tested sample was a polysaccharide substance [38]. The peak at 1734 cm^−1^ was attributed to the stretching vibration of C=O, a characteristic of carboxylic acid esters, indicating that CSPs might be an acidic polysaccharide [27]. The absorption peak at 1418 cm^−1^ was attributed to stretching and bending vibrations of C-H, while that at 1598 cm^−1^ was attributed to C=O asymmetric stretching vibration, suggesting the existence of uronic acid in CSPs [39]. The peak at 1010 cm^−1^ indicated the presence of the pyran rings in CSPs. The characteristic absorption peaks at 914 cm^−1^ and 833 cm^−1^ indicated the presence of β-glycosidic and α-glycosidic bonds, respectively [33]. FT-IR results suggested that CSPs might be acidic heteropolysaccharides containing pyran rings, α- and β-glycosidic bonds and glucuronic acid, bearing similarity to the polysaccharides from coriander seeds or fresh coriander [19,20].

### 3.5. Monosaccharide Composition Analysis of CSP

**Figure 4:**
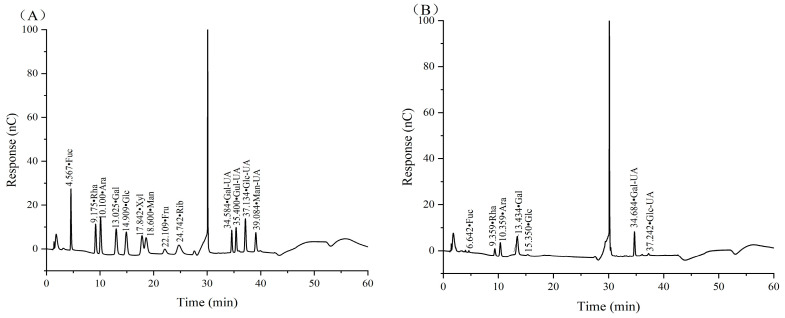
Ion chromatograms of standard monosaccharides (**A**) and CSPs (**B**).

The monosaccharide composition, as well as glycosidic bond type, is one of the main structural characteristics of polysaccharides and is closely related to their biological activity. According to the retention time of the mixed standard monosaccharides, CSPs were mainly composed of fucose (Fuc), rhamnose (Rha), arabinose (Ara), galactose (Gal), glucose (Glc), galacturonic acid (Gal UA) and glucuronic acid (Glc UA), with a molar ratio of 1.13:15.11:9.60:25.98:1.55:44.33:2.29 (Figure 4). It was clear that Gal UA and Gal occupied the largest and second largest molar ratio of monosaccharides in CSPs.

The differences in monosaccharide composition between our CSPs and the coriander polysaccharides in references [19,20] were the composition and molar ratio of monosaccharides. In reference [19], Gal and Glc occupied the largest and second largest ratio, while Man, which was not detected in our paper and reference [20], occupied the third largest ratio. The difference might be because it was extracted from coriander seeds while the other two were extracted from fresh coriander. In reference [20], Gal and Ara occupied the largest and second largest molar ratio, which was 20.85 and 8.14, respectively, while Gal UA accounted for just 2.42. The difference might be because CSPs were extracted using 240 W ultrasonic-assisted hot water, and sonication may have led to an increase in Gal UA content as Gal UA was more resistant to sonication compared to neutral sugars [40].

### 3.6. DPPH and ABTS Antioxidant Activity of CSP

**Figure 5:**
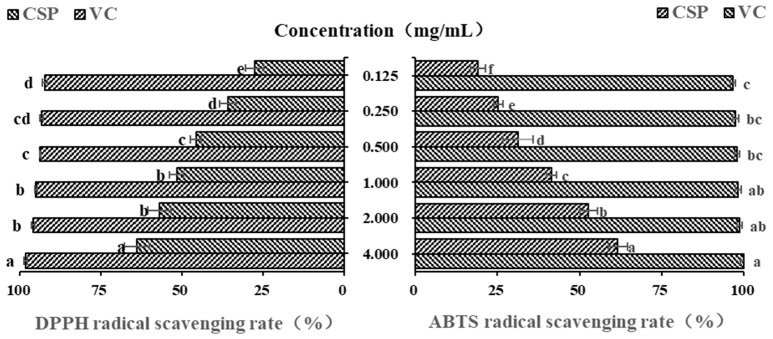
In vitro DPPH and ABTS antioxidant activity of CSPs. Vc was used as a positive control. Different lowercase letters represent significant difference at 0.05 level.

Polysaccharides from plants are often considered and explored as potential antioxidants and free radical scavengers [41]. Due to their low cost, high efficiency and simple operation, DPPH and ABTS free radicals are often the first choice for evaluating antioxidant activity of natural compounds [42,43]. Vc is undoubtedly a strong antioxidant, and it is often used as a positive control in antioxidant activity experiments [44]. In this study, as shown in Figure 5, within the concentration range of 0.125–1.00 mg/mL, the DPPH free radical scavenging ability of CSPs gradually increased with their concentration (*p* < 0.05). However, there was no significant difference in DPPH free radical scavenging ability between 1.0 mg/mL and 2.0 mg/mL. When the concentration was 4 mg/mL, CSPs showed a much higher DPPH scavenging rate (63.37 ± 3.84%), which was a little lower than that of polysaccharides from lychee nuclear (66.67%) [31] but higher than that of the previous reported polysaccharide content from coriander (about 50–60% when at a concentration of 7.5 mg/mL) [20]. The half maximal inhibitory concentration (IC_50_) of CSPs was 0.759 mg/mL. The results showed that CSPs had a strong scavenging property on DPPH free radicals.

The newly prepared ABTS free radical solution appears blue-green in color and exhibits typical ultraviolet absorption at 734 nm. When ABTS free radical pairs with electrons or hydrogen provided by antioxidants, the absorbance will decrease proportionally and the solution will exhibit a lighter color, which can be used to evaluate the antioxidant capacity [37,41]. Figure 5 showed that within the concentration range of 0.125~4.0 mg/mL, the ABTS free radical scavenging ability of CSPs showed significant concentration dependence (*p* < 0.05), and the IC_50_ of CSPs was 1.758 mg/mL. The ABTS free radical scavenging rate of 4 mg/mL of CSPs was 61.67 ± 0.30%, indicating a strong ability to donate electrons or hydrogen [45].

### 3.7. In Vitro Hypoglycemic Activity of CSP

**Figure 6:**
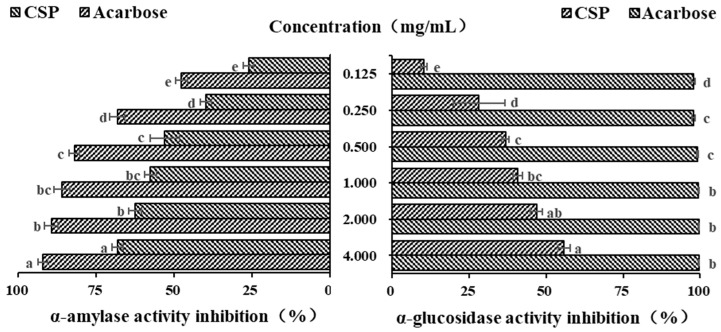
In vitro hypoglycemic activity of CSPs. Acarbose was used as a reference. Different lowercase letters represent significant difference at 0.05 level.

α-amylase, as a key starch degrading enzyme, can rapidly degrade starch into glucose, leading to a sharp increase in blood sugar levels. Polysaccharides can effectively slow down the rate of glucose production and reduce and delay the absorption of glucose by the intestine, thereby effectively reducing blood sugar levels [34]. Acarbose, originating from microbes, is a commonly used drug to treat type II diabetes. In the current study, it was used as a positive control. As shown in Figure 6, within the concentration range of 0.125–0.5 mg/mL, the inhibitory ability of both acarbose and CSPs on α-amylase increased in a concentration-dependent manner (*p* < 0.05). When the concentration increased from 0.5 to 2.0 mg/mL, the increasing inhibitory abilities of acarbose and CSPs on α-amylase both began to plateau. When the concentration was 4 mg/mL, the inhibition rates of acarbose and CSPs on α-amylase were 92.25 ± 1.51% and 68.20 ± 1.72%, respectively. The IC_50_ of acarbose and CSPs for α-amylase were 0.108 and 0.634 mg/mL, respectively. It was reported that the IC_50_ values of polysaccharides from *Ribes nigrum* and *Rosa roxburghii* was 6.26 and 4.77 mg/mL, respectively, when the IC_50_ values of acarbose were 0.34 and 0.01185 mg/mL, respectively [34,46]. The above results showed that polysaccharides from different plants showed different hypoglycemic activity, and CSPs have a stronger inhibition effect on α-amylase.

α-glucosidase is widely used in in vitro hypoglycemic experiments [45]. As an important enzyme in the glucose metabolism pathway, it is mainly distributed on the brush edge of the small intestinal epithelial villi and can act on glucosidic bonds, hydrolyzing carbohydrate substances into glucose as the final product. PNPG is often used as a substrate to evaluate the inhibitory ability of α-glucosidase inhibitors. As shown in Figure 6, the inhibitory ability of CSPs on α-glucosidase was much lower than that of acarbose, and it increased significantly in a dose-dependent manner, with the strongest upward trend in the concentration range of 0.125–0.500 mg/mL (*p* < 0.05). When the concentration reached 1.000 mg/mL, the inhibition rate of growth slowed down, which was similar to *Fagopyrum tartaricum* polysaccharides (when the concentration reached 0.8 mg/mL) [29]. The maximum inhibition rate of CSPs at a concentration of 4.0 mg/mL was 55.83 ± 1.82%, and that of acarbose at the same concentration was 99.69 ± 0.21%. The IC_50_ of CSPs for α-glucosidase was 2.178 mg/mL, which was lower than that of polysaccharides from Ribes nigrum (4.32 mg/mL) [30], indicating a stronger inhibition ability to α-glucosidase. Many natural polysaccharides have been shown to have good inhibition effects on α-glucosidase activity, although the inhibition ability was lower than that of acarbose [46,47]. The results of both α-amylase and α-glucosidase showed that CSPs have the potential to control blood sugar levels as functional food.

### 3.8. In Vitro Immunomodulatory Activity of CSPs

#### 3.8.1. Effects of CSPs on RAW264.7 Cell Viability

**Figure 7:**
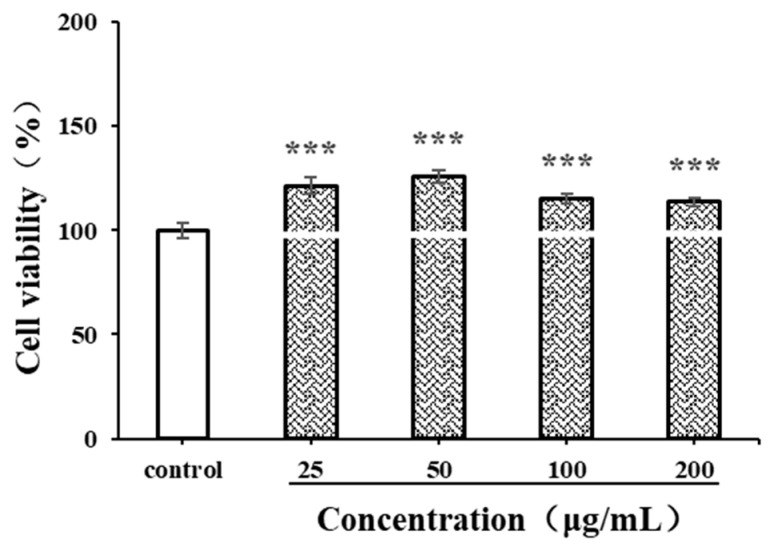
Effects of CSPs on RAW264.7 cytotoxicity. Compared with the control group, *** represents *p* < 0.001.

Before evaluating immunomodulatory activity, the cytotoxicity of antioxidants on macrophages should be detected. The CCK-8 method is used to investigate the viability of live cells by measuring the activity of mitochondrial dehydrogenase [48]. LPSs, produced by *Escherichia coli* O55:B5, are often used as a positive control in macrophage-activating experiments. In the current study, the effects of CSPs and LPSs (1 μg/mL) on the proliferation of RAW264.7 cells were detected. As shown in Figure 7, within the concentration range of 25–200 μg/mL, CSPs showed no significant cytotoxicity to RAW264.7 cells (*p* < 0.001). The proliferation of RAW264.7 cells exhibited a pattern of initial increase followed by decrease, with an increasing CSP concentration (25–200 μg/mL). Within the concentration range of 25–50 μg/mL, the proliferation of RAW264.7 cells increased with the concentration (*p* < 0.001). Then, the proliferation rate began to decrease; although within the concentration range of 50–200 μg/mL, it still significantly promoted the proliferation compared to the control group (*p* < 0.001). The results showed that CSPs had a good macrophage activation capability.

#### 3.8.2. Effects of CSPs on Phagocytosis of RAW264.7 Cells

**Figure 8:**
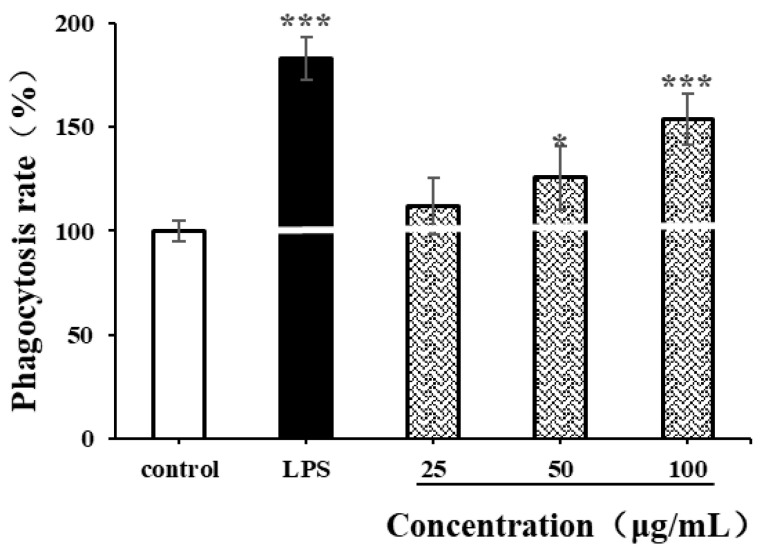
Effects of CSPs on phagocytosis of RAW264.7 cells. Compared with the negative control group, * represents *p* < 0.05 and *** represents *p* < 0.001.

The phagocytic characteristics of macrophages are some of the indicators for measuring the immunomodulatory activity of natural polysaccharides [35,49]. In our study, neutral red assay was used to determine the effects of LPSs (1 µg/mL) and CSPs on the phagocytic ability of RAW264.7 cells. As shown in Figure 8, LPSs significantly enhanced the phagocytic ability of RAW264.7 cells (*p* < 0.001), while CSPs had varying degrees of enhancement on RAW264.7 cells within the concentration range of 25–100 µg/mL. Within the concentration range of 50–100 μg/mL, the proliferation of RAW264.7 cell increased with the concentration (*p* < 0.001). When the concentration reached 100 µg/mL, there was a significant difference in cell phagocytic ability compared to the control group (*p* < 0.001), with a phagocytic rate of 153.75 ± 12.05%. The results showed that CSPs could activate the phagocytosis of macrophages, which was similar to polysaccharides from *Collybia radicata* and *Asparagus officinalis* [35,49].

#### 3.8.3. Effects of CSPs on NO Secretion in RAW264.7 Cells

**Figure 9:**
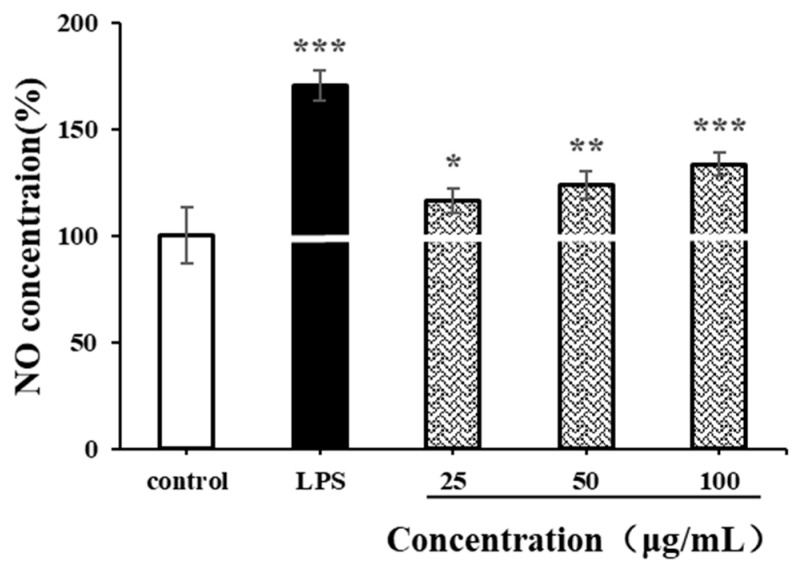
Effects of CSPs on NO secretion in RAW264.7 cells. Compared with the blank control group, * represents *p* < 0.05, ** represents *p* < 0.01, and *** represents *p* < 0.001.

NO is an important biological messenger associated with physiological functions of the cardiovascular, immune and nervous systems [49]. As shown in Figure 9, the NO secretion level of RAW264.7 cells significantly increased after LPS stimulation (*p* < 0.001). Compared with the negative control group, CSPs could also promote the production of NO in RAW264.7 cells at a low concentration of 25 µg/mL (*p* < 0.05). At a high concentration of 100 µg/mL, the production of NO increased continually (*p* < 0.001). The NO concentration was 1.33 times higher than that of the negative control and was 0.77 times lower than that of the positive control. The increase in NO concentration in activated RAW264.7 usually contributes to the in vitro immune response. The results indicated that CSPs had strong immunomodulatory by increasing cellular NO levels in a concentration-dependent manner within the range of 25–100 µg/mL, which was similar to polysaccharides from *Collybia radicata* and *Ganoderma lucidum* spores [48,49].

#### 3.8.4. Effects of CSPs on Proliferation of HepG2 Cells

**Figure 10:**
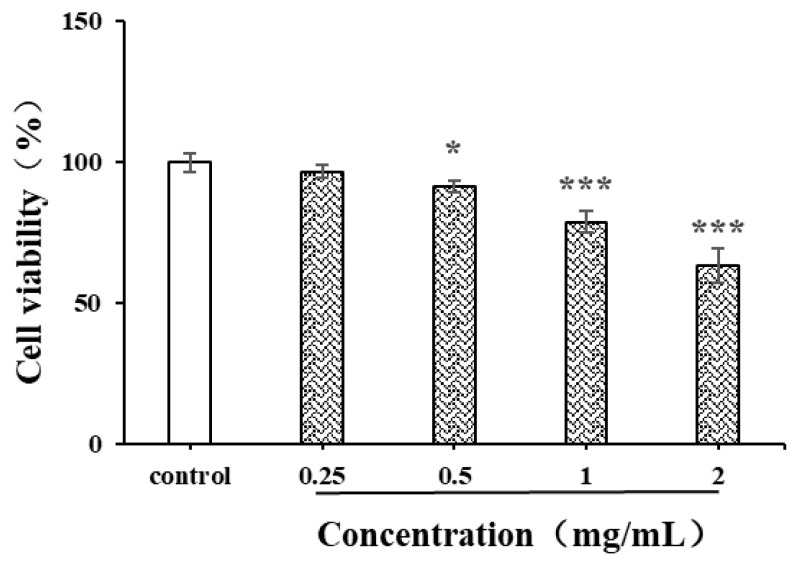
Effects of CSPs on proliferation of HepG2 cells. Compared with the control group, * represents *p* < 0.05 and *** represents *p* < 0.001.

Natural polysaccharides are reported to have strong anti-cancer activity due to the free radical scavenging capacity and inhibition capacity of tumor cells [41]. The liver cancer cell HepG2 was used in the current study to evaluate the in vitro anticancer activity of CSPs (Figure 10). After 24 h of treatment with CSPs, the proliferation ability of HepG2 cells gradually weakened with the increase in concentration and showed significant differences within the concentration range of 0.5–2 mg/mL. The inhibition rates of CSPs at 0.5, 1 and 2 mg/mL were 90.9, 78.2 and 63.6%, respectively. When the concentration exceeded 1.0 mg/mL, the inhibition of HepG2 cell viability became more significant (*p* < 0.001). Treated with the dandelion leaf polysaccharides, at a dose of 1 mg/mL, the HepG2 cell showed a cell viability of 29.4% [28], indicating a stronger potential than CSPs to inhibit the proliferation of HepG2 cells. The mechanism by which CSPs inhibited HepG2 cell viability might be similar to that of dandelion leaf polysaccharides, which induce cell apoptosis, indicating their potential as anticancer agents [28].

## 4. Conclusions

In this study, polysaccharides from the whole herb of fresh coriander were extracted, and their physicochemical and structural properties and biological activity were analyzed. The purity of CSPs was relatively high, with a total sugar content and protein content of 66.90 ± 1.44% and 1.06 ± 0.32%, respectively. The main three kinds of monosaccharides of CSPs were Gal UA, Gal and Rha. Structural analysis showed that CSPs might be acidic heteropolysaccharides containing pyran rings, α- and β-glycosidic bonds and glucuronic acid. The results of the activity study showed that coriander polysaccharides have good in vitro antioxidant activity, with IC_50_ values of 0.759 mg/mL and 1.758 mg/mL for DPPH and ABTS free radical scavenging ability, respectively. At the final concentration of 4 mg/mL in the experiment, the inhibition rates of coriander polysaccharides on α-amylase and α-glucosidase were 68.20 ± 1.72% and 55.83 ± 1.82%, respectively. It was found that coriander polysaccharides have strong immune regulatory activity, by increasing RAW264.7 cell viability, enhancing phagocytic activity and promoting NO secretion. In addition, they could also inhibit the proliferation of HepG2 liver cancer cells in a concentration-dependent manner. The research aimed to provide new ideas for the comprehensive development and utilization of coriander in medicine, food and other fields and provide theoretical support for the in-depth study of coriander polysaccharides.

## Figures and Tables

**Table 1:** DPPH radical scavenging assay system.

Reagent	A3	A1	A2
DPPH	1 mL	1 mL	0 mL
CSP or Vc	0 mL	2 mL	2 mL
anhydrous ethanol	2 mL	0 mL	1 mL

**Table 2:** ATBS radical scavenging assay system.

Reagent	A3	A1	A2
ABTS	2 mL	2 mL	0 mL
CSP or Vc	0 mL	0.5 mL	0.5 mL
PBS	0.5 mL	0 mL	2 mL

**Table 3:** Reaction systems for α-amylase inhibitory assay.

Reagent	A3	A1	A2
α-amylase	0.2 mL	0.2 mL	0
CSPs or acarbose	0	0.2 mL	0.2 mL
Be incubated at 37 °C for 10 min			
Soluble starch	0.2 mL	0.2 mL	0.2 mL
Be incubated at 37 °C for 10 min			
DNS	0.4 mL	0.4 mL	0.4 mL
Be boiling water bathed for 5 min, followed by cooling and then the addition of 5 mL of distilled water			

**Table 4:** Reaction systems for α-glucosidase inhibitory assay.

Reagent	A3	A1	A2
α-glucosidase	0.1 mL	0.1 mL	0
CSPs or acarbose	0	0.1 mL	0.1 mL
Be incubated at 37 °C for 10 min			
PNPG	0.1 mL	0.1 mL	0.1 mL
Be incubated at 37 °C for 15 min			
Sodium carbonate solution	0.2 mL	0.2 mL	0.2 mL

## Data Availability

Data are contained within the article. The original contributions presented in this study are included in the article. Further inquiries can be directed to the corresponding author.

## References

[1] Prachayasittikul V., Prachayasittikul S., Ruchirawat S., Prachayasittikul V. (2018). Coriander (*Coriandrum sativum*): A Promising Functional Food toward the Well-Being. Food Res. Int..

[2] Wei J.-N., Liu Z.-H., Zhao Y.-P., Zhao L.-L., Xue T.-K., Lan Q.-K. (2019). Phytochemical and Bioactive Profile of *Coriandrum sativum* L.. Food Chem..

[3] Wei S., Wei L., Xie B., Li J., Lyu J., Wang S., Khan M.A., Xiao X., Yu J. (2024). Characterization of Volatile Profile from Different Coriander (*Coriandrum sativum* L.) Varieties via HS-SPME/GC–MS Combined with E-Nose Analyzed by Chemometrics. Food Chem..

[4] Spence C. (2023). Coriander (Cilantro): A Most Divisive Herb. Int. J. Gastron. Food Sci..

[5] Adams M., Gmünder F., Hamburger M. (2007). Plants Traditionally Used in Age Related Brain Disorders—A Survey of Ethnobotanical Literature. J. Ethnopharmacol..

[6] Salem M.A., Manaa E.G., Osama N., Aborehab N.M., Ragab M.F., Haggag Y.A., Ibrahim M.T., Hamdan D.I. (2022). Coriander (*Coriandrum sativum* L.) Essential Oil and Oil-Loaded Nano-Formulations as an Anti-Aging Potentiality via TGFβ/SMAD Pathway. Sci. Rep..

[7] Attaallah A., Elmrazeky A.R., El-Beltagy A.E.-F.B.M., Abdelaziz K.K., Soliman M.F. (2023). Modulatory Role of *Coriandrum sativum* (Coriander) Extract against Diabetic Complications on the Gonads of Female Rats and Their Offspring. Tissue Cell.

[8] Mechchate H., Es-safi I., Amaghnouje A., Boukhira S., Alotaibi A.A., Al-zharani M., A. Nasr F., M. Noman O., Conte R., Amal E.H.E.Y. (2021). Antioxidant, Anti-Inflammatory and Antidiabetic Proprieties of LC-MS/MS Identified Polyphenols from Coriander Seeds. Molecules.

[9] Ohara R., Sugahara T., Sugie Y., Onda H., Yoshino N., Nishi K., Ishida M., Kikuzaki H. (2022). Identification of Components in Coriander (*Coriandrum sativum* L.) Inhibiting Degranulation of RBL-2H3 Cells. Fitoterapia.

[10] Yu Y., Cheng Y., Wang C., Huang S., Lei Y., Huang M., Zhang X. (2023). Inhibitory Effect of Coriander (*Coriandrum sativum* L.) Extract Marinades on the Formation of Polycyclic Aromatic Hydrocarbons in Roasted Duck Wings. Food Sci. Hum. Wellness.

[11] Karaca N., Demirci F. (2024). In Vitro Cholinesterase, Lipoxygenase Inhibition Evaluation of Rosemary and Coriander Essential Oil Nanoemulsion and Characterisation. J. Herb. Med..

[12] Ghazanfari N., Tabatabaei Yazdi F., Mortazavi S.A., Mohammadi M. (2023). Using Pulsed Electric Field Pre-Treatment to Optimize Coriander Seeds Essential Oil Extraction and Evaluate Antimicrobial Properties, Antioxidant Activity, and Essential Oil Compositions. LWT.

[13] Mahmoud M.F., Ali N., Mahdi I., Mouhtady O., Mostafa I., El-Shazly A.M., Abdelfattah M.A.O., Hasan R.A., Sobeh M. (2023). Coriander Essential Oil Attenuates Dexamethasone-Induced Acute Liver Injury through Potentiating Nrf2/HO-1 and Ameliorating Apoptotic Signaling. J. Funct. Foods.

[14] Kačániová M., Galovičová L., Ivanišová E., Vukovic N.L., Štefániková J., Valková V., Borotová P., Žiarovská J., Terentjeva M., Felšöciová S. (2020). Antioxidant, Antimicrobial and Antibiofilm Activity of Coriander (*Coriandrum sativum* L.) Essential Oil for Its Application in Foods. Foods.

[15] Ghazanfari N., Mortazavi S.A., Yazdi F.T., Mohammadi M. (2020). Microwave-Assisted Hydrodistillation Extraction of Essential Oil from Coriander Seeds and Evaluation of Their Composition, Antioxidant and Antimicrobial Activity. Heliyon.

[16] Kajal A. (2018). An Allied Approach for in Vitro Modulation of Aldose Reductase, Sorbitol Accumulation and Advanced Glycation End Products by Flavonoid Rich Extract of *Coriandrum sativum* L. Seeds. Toxicol. Rep..

[17] Scandar S., Zadra C., Marcotullio M.C. (2023). Coriander (*Coriandrum sativum*) Polyphenols and Their Nutraceutical Value against Obesity and Metabolic Syndrome. Molecules.

[18] Song E.-J., Ko M.-J. (2022). Extraction of Monoterpenes from Coriander (*Coriandrum sativum* L.) Seeds Using Subcritical Water Extraction (SWE) Technique. J. Supercrit. Fluids.

[19] Sfar M., Souid G., Alminderej F.M., Mzoughi Z., El-Ghoul Y., Rihouey C., Le Cerf D., Majdoub H. (2023). Structural Characterization of Polysaccharides from *Coriandrum sativum* Seeds: Hepatoprotective Effect against Cadmium Toxicity In Vivo. Antioxidants.

[20] Chang M., Shi S., Liu H., Tu J., Yan Z., Ding S. (2022). Extraction, Characterization, and in vivo Antitumor Activity of a Novel Polysaccharide from *Coriandrum sativum* L.. J. Food Biochem..

[21] Liu Y., Shi Y., Zou J., Zhang X., Zhai B., Guo D., Sun J., Luan F. (2024). Extraction, Purification, Structural Features, Biological Activities, Modifications, and Applications from *Taraxacum Mongolicum* Polysaccharides: A Review. Int. J. Biol. Macromol..

[22] Liu P., Fei L., Wu D., Zhang Z., Chen W., Li W., Yang Y. (2024). Progress in the Metabolic Kinetics and Health Benefits of Functional Polysaccharides from Plants, Animals and Microbes: A Review. Carbohydr. Polym. Technol. Appl..

[23] Fernandes P.A.R., Coimbra M.A. (2023). The Antioxidant Activity of Polysaccharides: A Structure-Function Relationship Overview. Carbohydr. Polym..

[24] Liu Y., Sun Y., Huang G. (2018). Preparation and Antioxidant Activities of Important Traditional Plant Polysaccharides. Int. J. Biol. Macromol..

[25] Yin M., Zhang Y., Li H. (2019). Advances in Research on Immunoregulation of Macrophages by Plant Polysaccharides. Front. Immunol..

[26] Wang J., Zhang A., Hu Y., Yuan X., Qiu Y., Dong C. (2024). Polysaccharides from Fructus Corni: Extraction, Purification, Structural Features, and Biological Activities. Carbohydr. Res..

[27] Chen N., Ding Y., Li X., Li J., Cheng Y., Tian Y., Tian Y., Wu M. (2024). Chemical Structures and Immunomodulatory Activities of Polysaccharides from *Polygonatum kingianum*. Int. J. Biol. Macromol..

[28] Chen P., Ding S., Yan Z., Liu H., Tu J., Chen Y., Zhang X. (2023). Structural Characteristic and In-Vitro Anticancer Activities of Dandelion Leaf Polysaccharides from Pressurized Hot Water Extraction. Nutrients.

[29] Wang X.-T., Zhu Z.-Y., Zhao L., Sun H.-Q., Meng M., Zhang J.-Y., Zhang Y.-M. (2016). Structural Characterization and Inhibition on α-d-Glucosidase Activity of Non-Starch Polysaccharides from *Fagopyrum tartaricum*. Carbohydr. Polym..

[30] Yang W., Huang G. (2023). Preparation and Analysis of Polysaccharide from *Solanum tuberdsm*. Ultrason. Sonochem..

[31] Song Z., Huang G., Huang H. (2024). The Ultrasonic-Assisted Enzymatic Extraction, Characteristics and Antioxidant Activities of Lychee Nuclear Polysaccharide. Ultrason. Sonochem..

[32] DuBois M., Gilles K.A., Hamilton J.K., Rebers P.A., Smith F. (1956). Colorimetric Method for Determination of Sugars and Related Substances. Anal. Chem..

[33] Que Y., Zhang Y., Liang F., Wang L., Yang Y., Zhang J., Wang W., Sun Y., Zhong C., Zhang H. (2024). Structural Characterization, Antioxidant Activity, and Fermentation Characteristics of *Flammulina velutipes* Residue Polysaccharide Degraded by Ultrasonic Assisted H_2_O_2_-Vc Technique. Ultrason. Sonochem..

[34] Zhao M., Bai J., Bu X., Yin Y., Wang L., Yang Y., Xu Y. (2021). Characterization of Selenized Polysaccharides from *Ribes Nigrum* L. and Its Inhibitory Effects on α-Amylase and α-Glucosidase. Carbohydr. Polym..

[35] Wang N., Zhang X., Wang S., Guo Q., Li Z., Liu H., Wang C. (2020). Structural Characterisation and Immunomodulatory Activity of Polysaccharides from White Asparagus Skin. Carbohydr. Polym..

[36] Wang H., Huang G. (2024). Extraction, Purification, Structural Modification, Activities and Application of Polysaccharides from Different Parts of *Mulberry*. Food Funct..

[37] Li G., Chen P., Zhao Y., Zeng Q., Ou S., Zhang Y., Wang P., Chen N., Ou J. (2021). Isolation, Structural Characterization and Anti-Oxidant Activity of a Novel Polysaccharide from *Garlic bolt*. Carbohydr. Polym..

[38] Li Y.-M., Zhong R., Chen J., Luo Z.-G. (2021). Structural Characterization, Anticancer, Hypoglycemia and Immune Activities of Polysaccharides from *Russula virescens*. Int. J. Biol. Macromol..

[39] He Y., Peng H., Zhang H., Liu Y., Sun H. (2021). Structural Characteristics and Immunopotentiation Activity of Two Polysaccharides from the Petal of *Crocus sativus*. Int. J. Biol. Macromol..

[40] Ogutu F.O., Mu T.-H. (2017). Ultrasonic Degradation of Sweet Potato Pectin and Its Antioxidant Activity. Ultrason. Sonochem..

[41] Patel M.K., Tanna B., Mishra A., Jha B. (2018). Physicochemical Characterization, Antioxidant and Anti-Proliferative Activities of a Polysaccharide Extracted from Psyllium (*P. ovata*) Leaves. Int. J. Biol. Macromol..

[42] Wołosiak R., Drużyńska B., Derewiaka D., Piecyk M., Majewska E., Ciecierska M., Worobiej E., Pakosz P. (2022). Verification of the Conditions for Determination of Antioxidant Activity by ABTS and DPPH Assays—A Practical Approach. Molecules.

[43] Cai L., Zou S., Liang D., Luan L. (2018). Structural Characterization, Antioxidant and Hepatoprotective Activities of Polysaccharides from *Sophorae tonkinensis* Radix. Carbohydr. Polym..

[44] Peng J., Hu T., Li J., Du J., Zhu K., Cheng B., Li K. (2019). Shepherd’s Purse Polyphenols Exert Its Anti-Inflammatory and Antioxidative Effects Associated with Suppressing MAPK and NF-*κ*B Pathways and Heme Oxygenase-1 Activation. Oxidative Med. Cell. Longev..

[45] Ren B., Chen C., Li C., Fu X., You L., Liu R.H. (2017). Optimization of Microwave-Assisted Extraction of *Sargassum thunbergii* Polysaccharides and Its Antioxidant and Hypoglycemic Activities. Carbohydr. Polym..

[46] Wang L., Zhang B., Xiao J., Huang Q., Li C., Fu X. (2018). Physicochemical, Functional, and Biological Properties of Water-Soluble Polysaccharides from *Rosa roxburghii Tratt* Fruit. Food Chem..

[47] Yang Y., Lei Z., Zhao M., Wu C., Wang L., Xu Y. (2020). Microwave-Assisted Extraction of an Acidic Polysaccharide from *Ribes nigrum* L.: Structural Characteristics and Biological Activities. Ind. Crops Prod..

[48] Sheng Z., Wen L., Yang B. (2022). Structure Identification of a Polysaccharide in Mushroom Lingzhi Spore and Its Immunomodulatory Activity. Carbohydr. Polym..

[49] Wang Y., Tian Y., Shao J., Shu X., Jia J., Ren X., Guan Y. (2018). Macrophage Immunomodulatory Activity of the Polysaccharide Isolated from *Collybia radicata* Mushroom. Int. J. Biol. Macromol..

